# The Role of Extracellular Proteases in Tumor Progression and the Development of Innovative Metal Ion Chelators That Inhibit Their Activity

**DOI:** 10.3390/ijms21186805

**Published:** 2020-09-16

**Authors:** Kyung Chan Park, Mahendiran Dharmasivam, Des R. Richardson

**Affiliations:** 1 Molecular Pharmacology and Pathology Program, Department of Pathology and Bosch Institute, Medical Foundation Building, University of Sydney, Sydney 2006, Australia; kpar.kyungchan@gmail.com (K.C.P.); magichem007@gmail.com (M.D.); 2 Centre for Cancer Cell Biology and Drug Discovery, Griffith Institute of Drug Discovery, Griffith University, Nathan, Brisbane 4111, Australia; 3 Department of Pathology and Biological Responses, Nagoya University Graduate School of Medicine, Nagoya 466-8550, Japan

**Keywords:** cancer therapeutics, thiosemicarbazones, prostate specific antigen, matrix metalloproteases

## Abstract

The role of extracellular proteases in cancer progression is well-known, especially in relation to the promotion of cell invasion through extracellular matrix remodeling. This also occurs by the ability of extracellular proteases to induce the shedding of transmembrane proteins at the plasma membrane surface or within extracellular vesicles. This process results in the regulation of key signaling pathways by the modulation of kinases, e.g., the epidermal growth factor receptor (EGFR). Considering their regulatory roles in cancer, therapeutics targeting various extracellular proteases have been discovered. These include the metal-binding agents di-2-pyridylketone 4,4-dimethyl-3-thiosemicarbazone (Dp44mT) and di-2-pyridylketone-4-cyclohexyl-4-methyl-3-thiosemicarbazone (DpC), which increase c-MET degradation by multiple mechanisms. Both the direct and indirect inhibition of protease expression and activity can be achieved through metal ion depletion. Considering direct mechanisms, chelators can bind zinc(II) that plays a catalytic role in enzyme activity. In terms of indirect mechanisms, Dp44mT and DpC potently suppress the expression of the kallikrein-related peptidase—a prostate-specific antigen—in prostate cancer cells. The mechanism of this activity involves promotion of the degradation of the androgen receptor. Additional suppressive mechanisms of Dp44mT and DpC on matrix metalloproteases (MMPs) relate to their ability to up-regulate the metastasis suppressors N-myc downstream regulated gene-1 (NDRG1) and NDRG2, which down-regulate MMPs that are crucial for cancer cell invasion.

## 1. Introduction

Extracellular proteases are essential in the regulation of myriads of physiological processes [1]. In fact, there are 69 known families of human proteases [2], including matrix metalloproteases (MMPs), serine proteases, and cysteine proteases that regulate different biological processes [3]. Some of the canonical roles of extracellular proteases have been known for many decades and include the digestion of consumed proteins by trypsin in the small intestine, while the relatively recent finding of proteases on the surface of exosomes further opens the door to understanding the complex biology of cell matrix remodeling by extracellular proteases [4].

Like many other proteins that are essential for normal physiology, proteases can also be deregulated, contributing to the formation and progression of various diseases, including cancer, and as such, can be therapeutically targeted [1,5]. Of note, the pivotal roles of extracellular proteases in the development of tumor cell invasion and metastasis have been comprehensively investigated and documented [6]. However, more recently, studies have revealed that proteases have complex roles in cancer, such as the inhibition of receptor tyrosine kinase signaling mediated by ectodomain shedding [7]. This review will discuss oncogenic functions that extracellular proteases mediate in cancer, as well as novel protease-targeting therapeutics currently in development. These include novel thiosemicarbazones from our laboratory [8,9,10].

## 2. Functions of Extracellular Proteases in Cancer Progression

The association of proteolytic enzymes with cancer is well established, with different proteolytic families, including serine proteases, plasmin, plasminogen activators, human tissue kallikreins and MMPs being involved in tumor growth, invasion, angiogenesis and metastasis [11,12]. In addition, a trypsin-like protease was identified in various cancers known as tumor-associated trypsinogen (TAT) [12], which induces a metastatic phenotype of cancer cells [12,13]. Furthermore, a group of proteases known as the a disintegrin and metalloproteinases (ADAMs), are known to shed membrane-bound proteins, such as receptor tyrosine kinases [14,15] and G protein-coupled receptors [16] to either mediate activation or inhibition of their associated signaling pathways [7]. Thus, diverse extracellular proteases play integral roles in cancer progression and metastasis [17]. In the sections below, key extracellular proteases and their mechanisms of release from cells are discussed, along with a description of inhibitors, particularly zinc(II)-binding ligands that demonstrate therapeutic promise.

## 3. MMPs: Regulation and Function in Cancer

The extracellular matrix is composed of a complex variety of biomolecules, serves a number of functions, and undergoes controlled remodeling for physiological processes, such as bone development, angiogenesis, and wound repair [18]. For cancer cells, the extracellular matrix is also a physical barrier, which prevents them from migrating into blood or lymph vessels to mediate metastasis [19]. Therefore, deregulated extracellular matrix degradation by increased MMP activity facilitates the effective invasion of tumor cells [20]. Furthermore, with uncontrolled remodeling of the extracellular matrix, angiogenesis occurs more easily, leading to increased tumor growth and metastasis [21,22].

The MMPs belong to a family of zinc(II)- and calcium(II)-dependent endopeptidases that cleave extracellular matrix proteins. To date, over 20 MMPs have been discovered and can be loosely classified based on their substrate specificity as collagenases, gelatinases, stromelysins, membrane-type MMPs (MT-MMPs), etc. [23,24]. The MMPs are secreted from cells as inactive zymogens and are found anchored to the plasma membrane or within the extracellular matrix [25]. The activation of MMPs occurs via proteolysis of the pro-peptide domain by other MMPs or by furin-like serine proteases [23,26]. Importantly, the activity of MMPs is constitutively regulated by endogenous protease inhibitors called tissue inhibitors of metalloproteinases (TIMPs). There are four known TIMPs, namely TIMP-1, -2, -3, and -4, with these inhibitors being capable of controlling the proteolytic activity of all known MMPs [25,27,28,29].

Of the various extracellular proteases, MMPs play a major role in extracellular matrix degradation [30]. The MMPs cleave diverse components of extracellular matrix, such as laminin, fibronectin, elastin, collagen and aggrecan [31]. A study by Gobin et al. analyzed 24 MMP proteases in 15 different cancer-types and demonstrated that there is dysregulated expression of MMPs in tumors compared to normal tissue. Considering dysregulated MMPs, MMP11 and MMP13 were the most significantly up-regulated in the tumors examined [32]. The correlation between MMP over-expression and metastasis has been demonstrated in numerous tumors in vitro and in vivo [33,34] including colorectal cancer [35], melanoma [36], lung carcinoma [36], and gastric carcinoma [37]. Furthermore, MMPs detected in cancer patient body fluids positively associate with adverse prognosis [38,39]. Imbalances of MMPs and their endogenous inhibitors, namely the TIMPs, leads to tumor angiogenesis and metastasis [40,41,42]. While MMPs are known to promote angiogenesis via diverse mechanisms (for a comprehensive review see [21]), a number of studies also revealed that some MMPs have antagonistic role in angiogenesis, generating anti-angiogenic factors, such as angiostatin [43] and tumstatin [44] via their proteolytic activity. Also, MMPs are known to cleave diverse extracellular substrates, including chemokines and cytokines, influencing the inflammatory response, cytokine-mediated cell signaling, etc. [45]. Furthermore, more recently, MMPs were identified in intracellular compartments, such as the cytoplasm and nucleus, where they are involved in various cellular processes (e.g., apoptosis) [46]. Thus, these findings show that MMPs have much more complex functions in cancer progression, which is a research area that requires further investigation. The diverse functions of various MMPs in cancer are described in Table 1.

**Figure 1:**
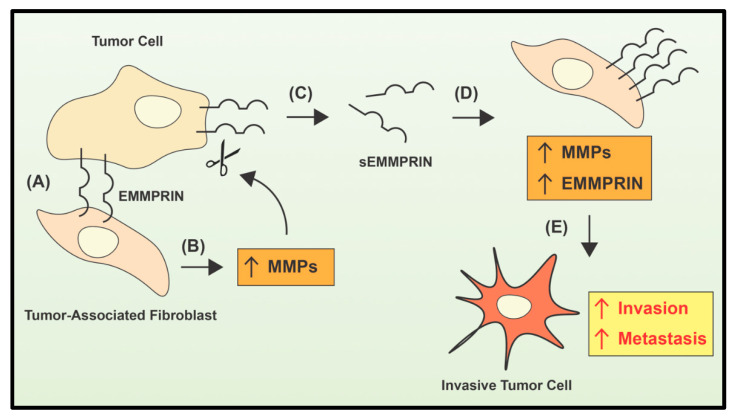
Interplay between EMMPRIN and MMP leads to metastasis. Soluble EMMPRIN (sEMMPRIN) and membrane-bound EMMPRIN form a bidirectional feedback between tumor cells and stromal cells. (**A**) Plasma membrane bound-EMMPRIN of the tumor cells interacts with cancer-associated stromal cells, such as fibroblasts. (**B**) This interaction between the cell-types induces up-regulation of MMP expression and secretion by the fibroblasts. (**C**) The MMPs secreted by fibroblasts cleave membrane-bound EMMPRIN to generate sEMMPRIN. (**D**) sEMMPRIN further augments MMP and EMMPRIN expression either in fibroblasts in the local tumor microenvironment or at distal sites. (**E**) This process then stimulates cancer cell invasion and metastasis. Modified from [56].

Intriguingly, in many solid tumors, MMPs are produced by tumor stromal cells, rather than by tumor cells, including tumor-associated interstitial collagenase (MMP-1), stromelysin-1 (MMP-3), stromelysin-3 (MMP-11), and gelatinase A (MMP-2) [47,48,49,50]. The induction of MMP production has been reported to occur, in part, via cancer cell-stromal cell interaction through the tumor cell surface protein, which is an extracellular matrix metalloproteinase inducer (EMMPRIN; [51,52]). EMMPRIN is a cell surface glycoprotein with a molecular weight of 58,000 Daltons and is a member of the immunoglobulin superfamily [53]. Apart from its membrane-bound form, a soluble form of EMMPRIN (sEMMPRIN) has also been identified and plays a key role in its biological function [54,55,56]. In fact, the soluble and membrane-bound forms of EMMPRIN enable bidirectional feedback between neoplastic cells and stromal cells, such as tumor-associated fibroblasts, leading to a positive feedback regulation of EMMPRIN expression and the MMP-dependent generation of soluble EMMPRIN [56]. In this latter model, cancer cells utilize plasma membrane-bound EMMPRIN to initiate contact with tumor stromal cells, such as fibroblasts (Figure 1) [56]. This signals to the fibroblasts to up-regulate MMP expression and secretion (Figure 1). The MMPs secreted by fibroblasts cleave cell surface EMMPRIN and generate soluble EMMPRIN (sEMMPRIN) that is then active in the local tumor environment, or can act on distal cells to further augment MMP and EMMPRIN expression to stimulate invasion and metastasis (Figure 1; [56]).

Therefore, the MMP group of extracellular proteases represent key targets for the development of new therapies for the treatment of cancer and these agents are described in greater detail in a subsequent section below (see Section 8).

## 4. Tumor-Associated Trypsinogen (TAT) and Kallikrein-Related Peptidases (KLKs) in Cancer

TAT was first identified in ovarian neoplasms [57]. Following its discovery, the expression of TAT was demonstrated in various cancers, including pancreatic cancer, hepatocellular carcinoma, cholangiocarcinomas, lung neoplasms, colorectal cancer, gastric cancer, colon carcinoma, fibrosarcoma, and erythroleukemia [58,59,60,61,62]. There are two isoforms of TAT, namely TAT-1 and TAT-2, with the latter being the predominant isoform [12].

TAT-2 and the pancreatic trypsin-2/anionic trypsin have an identical amino terminal sequence, molecular weight, and immunoreactivity, but differ in their activity against synthetic substrates, their enzyme stability, and their isoelectric point [63]. The level of TAT-2 in tumor cells has been correlated with the malignant phenotype and metastatic potential [12,13]. Furthermore, it has been demonstrated that TATs increase tumor growth by activating thrombin and plasminogen receptors, as well as other cell surface receptors, including growth factor receptors, integrins, and protease-activated receptor-2 (PAR-2; [64]). In particular, the activation of PAR-2 in MKN-1 gastric carcinoma cells has been demonstrated to regulate the adhesion and proliferation of these cells, while also stimulating cancer cell growth [64].

TAT-2, along with other proteases, such as MMPs, are also able to degrade the extracellular matrix, which makes cancer cells more invasive and aggressive [65]. In fact, TAT-2 activates pro-MMPs to begin protease cascades and was demonstrated to activate multiple pro-MMPs [63,66]. These include pro-MMP-1, -2, -3, -8, -9, and -13 and degrade type-I collagen, and all contribute to degradation of the extracellular matrix and promote tumor cell invasion and metastasis [63,66]. Therefore, TAT-2 can stimulate tumor growth and invasion via cleaving and activating cell surface receptors, MMPs, and degrading the extracellular matrix [67].

Kallikrein-related peptidases (KLKs) are serine proteases whose proteolytic activities regulate multiple physiological and pathological pathways [68]. There are 15 KLKs currently known [69] and in the context of cancer, these proteases are involved in the promotion of cellular proliferation, cancer cell survival, and invasion [68]. Of note, in prostate cancer, KLK-3, which is also commonly known as the prostate-specific antigen (PSA), has been used as a biomarker for prostate cancer screening, although its diagnostic value still remains controversial [68].

KLK-14 is another protease that is involved in prostate cancer progression, and its expression is elevated in metastatic and castration-resistant prostate cancer cells [70]. In pancreatic cancer, high KLK-7 expression is correlated with a poor prognosis [71], and silencing KLK-7 or the suppression of its activity with a small molecule inhibitor resulted in suppression of the proliferation, migration, and invasion of PANC-1 cells [72]. Furthermore, studies have shown that the majority of KLKs are aberrantly expressed in ovarian cancer compared to normal tissues [69]. In particular, KLK-6 [73] and KLK-10 [74] have demonstrated potential as clinically used serum biomarkers. Therefore, KLKs play vital roles in cancer progression in diverse tumor types.

## 5. Cross-Talk between Proteases and Kinases

Both proteases and kinases are responsible for the general processes of proteolysis and phosphorylation, respectively, with recent investigations demonstrating a complex interplay of activity that mediates many roles in physiology and pathology [75]. A variety of proteases are phosphorylated and dephosphorylated, regulating their biological activity, and many kinases are controlled by proteolysis, with these processes being integrated. This indicates a dynamic interplay of these two functions, which, when dysregulated, can contribute to the pathogenesis of cancer.

As a pertinent example of the interaction between proteases and kinases, ADAMs are engaged in protein ectodomain shedding, where these proteases act on many receptor tyrosine kinases, rapidly modifying their signaling effects, which can facilitate tumor invasion [76,77]. Due to the role of ADAMs in plasma membrane protein shedding, these enzymes are involved in the regulation of six out of the seven known ligands that affect expression and/or activation of the epidermal growth factor receptor (EGFR) tyrosine kinase, namely transforming growth factor-α (TGF-α), the epidermal growth factor (EGF), the heparin-binding epidermal growth factor (EGF)-like growth factor (HB-EGF), betacellulin, epiregulin, and amphiregulin [78].

Several studies have additionally demonstrated that cross-talk between G protein-coupled receptors and the epidermal growth factor receptor (EGFR) requires the activation of EGFR ligands by metalloproteases, including ADAM-10, ADAM-12, and ADAM-17 [79]. Considering these findings, and because the EGFR pathway is an important target for anti-cancer drug development [80], upstream activators of EGFR ligands, namely their sheddases, and regulators of these sheddases, could result in new druggable targets within the EGFR pathway [78,81].

Considering the regulation of sheddases, a number of ADAM-17-binding partners in the cytoplasm have been described, although their functions remain unclear [82,83,84]. However, considering the cross-talk between ADAM-17 and the EGFR, it is obvious that there is considerable complexity, with regulation of the cross-talk being critical between these proteins and many others. The reader is encouraged to examine the review by Blobel for a comprehensive analysis of this research area [77]. These facts indicate that extracellular proteases play far more complex roles than first imagined and continue to further justify attention in terms of their status as suitable targets for the development of novel therapeutics for cancer treatment.

## 6. The Proteases of Extracellular Vesicles (EVs) and Their Roles in Tumorigenesis

The sections above have detailed the key roles of extracellular proteases in invasion and oncogenesis and the potential role of shedding in regulating these processes. As an integral part of these processes in tumor biology, extracellular vesicles (EVs) have emerged as mediators of extracellular and intercellular communications in local and distant microenvironments, including distal organs [85,86,87]. It is well-known that EVs contain an extensive variety of critical biomolecules, such as proteins, DNA, RNA, and microRNAs, with recent studies demonstrating the presence of metalloproteinases, including cell surface-anchored sheddases, ADAMs and soluble ADAMTSs (ADAMs with thrombospondin motifs), and cell surface-bound and soluble MMPs [88].

The EV-associated metalloproteinases can alter the make-up of EVs by ectodomain shedding, with their contents also being capable of stimulating signaling pathways that could promote cancer progression or potentially inhibit it [87]. The bioactive cargo of EVs not only contains a variety of proteases, but also their regulators, such as extracellular matrix metalloproteinase inducers and TIMPs as novel means of matrix remodeling in physiological and pathological conditions [88].

Regarding the mechanisms responsible for the secretion of MMPs in EVs and their conversion into functionally active MMPs, this occurs by a distinct process to classical internalization mechanisms whereby membrane-bound MMPs are taken up via caveolae to form endosomes and then recycled to the plasma membrane [89]. As an example of the process of MMP secretion via EV formation, membrane type 1 matrix metalloproteinase (MT1-MMP) has been demonstrated in conditioned culture media taken from cells as microvesicular exosome cargo [90,91]. After its internalization into endosomes, MT1-MMP was not recycled back to the plasma membrane, but packaged into microvesicular exosomes and secreted into the extracellular environment, where it was functionally active [90]. These exosomes activated pro-MMP-2 and resulted in the degradation of type I collagen and gelatin, suggesting that exosomal MT1-MMP was functional.

It is of interest that activated platelet-derived microvesicles have been demonstrated to stimulate metastatic spread [92]. In these latter in vitro studies, platelet-derived microvesicles transferred the platelet integrin CD41 to lung cancer cells and enhanced MT1-MMP expression, while stimulating proliferation and invasion. Moreover, in vivo experiments examining the intravenous injection of platelet-derived microvesicle-treated Lewis lung carcinoma cells into syngeneic mice resulted in a significantly higher number of metastatic lung foci [92]. These studies provided evidence for the role of platelet-derived microvesicles in stimulating MT1-MMP in lung tumor cells and other mechanisms, resulting in enhanced metastasis [92].

It is well-known that the activity of MMPs leads to a loss of cellular adhesion and increased motility, facilitating the homing of tumor cells to new distal locations. Tumor cell-derived EVs stimulate MMP-9, IL-6, and transforming growth factor-β (TGF-β) and induce the secretion of EMMPRIN, which, in the tumor microenvironment, drives immune evasion, invasion, and inflammation that promote tumorigenesis [93]. In fact, this latter study suggested that EMMPRIN could be a general marker for secreted EVs, but also that EVs induce the secretion of full-length EMMPRIN from monocytes, which are particularly sensitive to these EVs. The up-regulation of MMP-9 by microvesicles is significant due to its role in extracellular matrix dissolution [94], which facilitates the breakdown of barriers that normally prevent cancer cells from invading distal sites [95]. The finding of increased EMMPRIN secretion with the associated increase of MMP-9 secretion suggests a higher metastatic potential, since their increased expression was correlated in several different cancers and is a poor prognostic indicator [96]. In fact, increased MMP-9 levels in cancer patient serum correlates with the probability of metastatic spread [97].

## 7. Therapeutics Targeting Proteases in Cancer

Considering the diverse roles that extracellular proteases play in cancer progression, anti-cancer drugs targeting proteases have been developed [98]. These protease inhibitors are either synthetic drugs or endogenous inhibitors [30]. The ADAMs are transmembrane proteases implicated in multiple processes, including cellular adhesion, cell fusion, cellular proliferation, and migration [99].

Of the 22 functional ADAMs expressed in humans, only half have MMP-like activity [99]. In contrast to MMPs that are involved in degradation of the extracellular matrix, the major ADAM substrates are the ectodomains of type-I and -II transmembrane proteins, such as growth factor or cytokine receptors [100]. Therefore, the ADAMs, particularly ADAM-10 and ADAM-17, have been described as the most prominent sheddases, being widely expressed and commonly over-expressed in cancer cells and involved in cleaving diverse substrates [7]. In the context of cancer, ADAM-10 and ADAM-17 have been indicated to be the principle proteases for EGFR ligands [78].

Several ADAMs, particularly ADAM-17, play functional roles in development, but also the progression of tumors. ADAM-17 is also known as a tumor necrosis factor-α (TNFα)-converting enzyme (TACE) and is now known to process more than 80 substrates [101]. Targeting ADAM-17 with either small molecular weight inhibitors or monoclonal antibodies has been demonstrated to induce anti-cancer activity [101]. A number of these have entered clinical trials. An example of one of these small molecular weight inhibitors of ADAM-17 is INCB7839 [102], which is a dual inhibitor of both ADAM-10 and ADAM-17, and has been trialed together with rituximab for the treatment of diffuse large B-cell non-Hodgkin lymphoma [103]. Moreover, INCB7839 has been tested in a clinical trial for the treatment of metastatic HER2+ breast cancer in combination with trastuzumab [104] and is currently in a phase 1 clinical trial for the treatment of recurrent/progressive high-grade gliomas (NCT04295759).

A significant challenge in developing novel small molecular weight drugs that inhibit ADAM-10 or -17 is the ability to selectively inhibit their pathological roles, while not targeting their key physiological processes, so as to prevent adverse effects. Certainly, the selectivity issue has been a major problem with the development of many small molecular weight inhibitors of ADAM-17 [101], with further studies and clinical trials being required. A potential solution could involve the use of antibodies. In fact, the antibody D1(A12) has been developed, which binds to the ADAM-17 catalytic and non-catalytic domains and demonstrates a sub-nanomolar IC_50_ [103]. This antibody also demonstrated selectivity for ADAM-17 and was not active up to 1000 nM against ADAM-10. Furthermore, D1(A12) prevented the shedding of cognate substrates of ADAM-17, such as TNF-α, TGF-β, etc. [103].

Regarding the many MMP inhibitors that have been designed, these can be divided into either (**1**) agents that directly ligate zinc(II), which forms a critical part of the catalytic active site, or (**2**) non-zinc(II)-binding agents that act to inhibit MMPs by a variety of mechanisms [105]. The mechanisms of action of the non-zinc(II)-binding MMP inhibitors have been thoroughly reviewed and will not be considered further here (see [105,106]). On the other hand, zinc(II)-ligating agents and the multiple mechanisms of inhibiting extracellular proteases mediated by drugs of the thiosemicarbazone class are described below in detail.

## 8. Thiosemicarbazones and Other Chelators Target Proteases by Indirect and Direct Mechanisms

### 8.1. Zinc(II) Chelators That Target MMPs

A number of studies have indicated that agents that bind metal ions, particularly zinc(II), can markedly affect the activity of a variety of extracellular proteases [105,106]. The key role of zinc(II) in metalloprotease activity and its ability to be ablated by chelators is underscored by the fact that the classical metal chelating ligand 1,10-phenanthroline, and to a lesser extent, EDTA, are used to inhibit metalloprotease activity [107,108]. These chelators act as relevant controls in biochemical gelatinolytic zymography assays assessing cancer cell gelatinase activity [107,108], but also gelatinase activity in normal human tissues and a variety of other organisms [109,110,111].

The MMP family is characterized by the presence of three conserved protein domains, namely (**1**) a pro-domain, (**2**) an active domain, and (**3**) a zinc(II)-binding domain [27]. The critical zinc(II)-binding domain consists of a conserved sequence composed of three histidine residues that coordinate zinc(II), which is catalytically vital for enzymatic activity [27]. In the pro-enzyme, a fourth cysteine residue ligates to zinc(II) and blocks enzymic activity. The pro-domain contains the critical cysteine residue and the catalytic domain contains the zinc(II)-binding site, which are the two domains common to all MMPs, with the activation of the enzyme being mediated through a “cysteine-switch” mechanism [112]. This process results in the loss of the pro-domain by proteolysis or substrate-binding [113].

**Figure 2:**
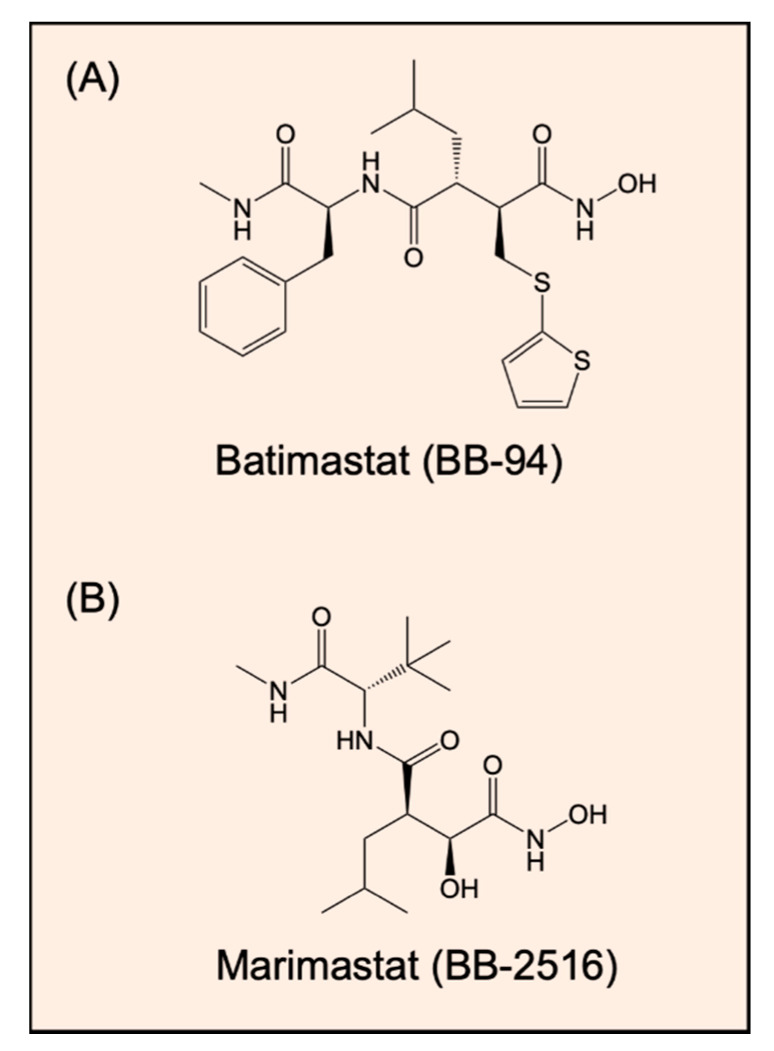
Line drawings of the structures of: (**A**) batimastat (BB-94) and (**B**) marimastat (BB-2516). These compounds are synthetic inhibitors of MMPs that act to potently inhibit these enzymes via their hydroxamate groups, which act to chelate zinc(II) that is required for MMP catalytic activity [106].

As additional examples of chelators as highly effective MMP inhibitors, batimastat (BB-94; Figure 2A) and marimastat (BB-2516; Figure 2B) are synthetic inhibitors of MMPs that act potently to inhibit these enzymes via their hydroxamate groups, which act to chelate zinc(II) that is required in the active site of MMPs [106]. In fact, batimastat demonstrated inhibitory activity in studies examining models of in vivo tumor growth and metastasis in mice [105,114] and was the first inhibitor of MMPs to be examined in humans with advanced malignancies [115]. However, the results from the early clinical trials of both batimastat and marimastat were disappointing. In fact, there were severe adverse side effects, such as the musculoskeletal syndrome, and moreover, there were no significant clinical benefits [116,117]. It has been suggested that the musculoskeletal toxicity was caused by the lack of selectivity of these broad-spectrum inhibitors, which prevented the activity of not only MMPs, but also non-MMP metalloproteinases, such as ADAMs and ADAMTs [118]. Again, this highlights the importance of selectivity in the development of MMP inhibitors in a similar manner to ADAM inhibitors. Furthermore, MMPs have complex roles in cancer progression, and these were not taken into account when the early clinical trials were designed and conducted for these broad-spectrum inhibitors [119].

Other commonly used pharmaceuticals that act as chelators also demonstrate activity against MMPs. For instance, tetracycline, doxycycline, and minocycline are members of the tetracycline group of antibiotics that bind divalent metal ions, including zinc(II) [120,121]. These drugs efficiently inhibit MMP-1, MMP-2, and MMP-12 in vitro and in vivo, with their ability to inhibit MMP-1 activity by 50% (IC_50_) being 15–350 µM [122]. The anthracycline antibiotics (daunorubicin, doxorubicin, and epirubicin), which act as metal ion chelators [123], can inhibit type IV collagenase activity and basement membrane degradation at IC_50_ values of 37–90 µM [124].

**Figure 3:**
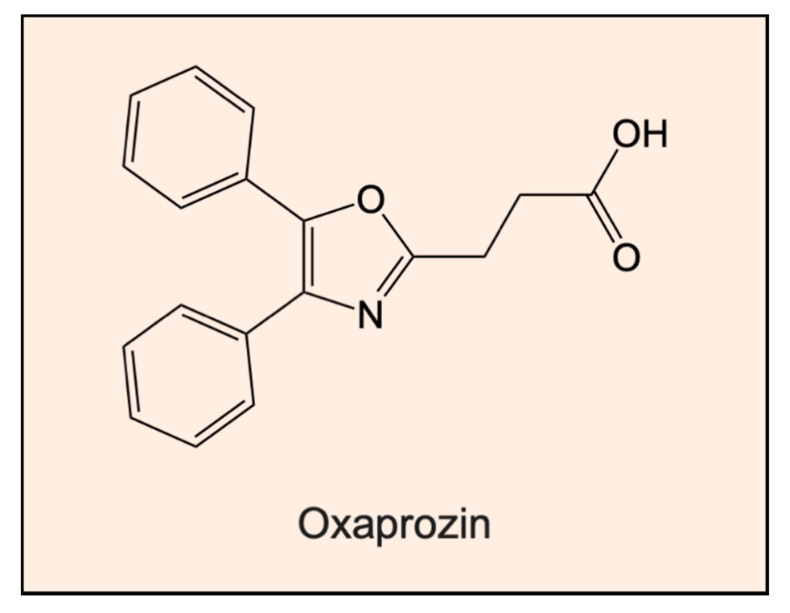
Line drawing of the structure of oxaprozin (4,5-diphenyl-2-oxazolepropionic acid). This agent is also a chelator and a non-steroidal, analgesic, and antipyretic propionic acid derivative that inhibits MMP9 activity by binding zinc(II) [125].

As a final example of another MMP inhibitor that is a chelator, it has been reported that oxaprozin (4,5-diphenyl-2-oxazolepropionic acid; Figure 3), which is a non-steroidal, analgesic, and antipyretic propionic acid derivative, inhibits MMP-9 activity by binding zinc(II) [125]. The chelation of zinc(II) by oxaprozin occurs through the nitrogen of its oxazole ring and the carboxylate moiety of the agent (Figure 3). Both gelatin zymography and enzymatic inhibition assays demonstrated that the inhibition observed was competitive in nature [125].

As noted by Jacobsen and colleagues [105], concern has been raised that the use of chelators, such as hydroxamic acids, may preclude the development of selective MMP inhibitors [126,127,128]. This could be due to undesired off-target effects on other metal-containing proteins [129]. As discussed above, selectivity is critical in developing effective ADAM or MMP inhibitors to ensure a low toxicity and high efficacy considering the diverse and complex functions of these metalloproteinases. One therapeutic approach that could resolve the issue of selectivity is utilizing antibody-based drugs, such as andecaliximab, which is a selective inhibitor of MMP-9 that has been examined in a number of clinical trials for the treatment of gastric/gastroesophageal junction adenocarcinoma (NCT01803282, NCT02864381, and NCT02545504) [130,131]. Number of hydroxamate- and carboxylate-based inhibitors of MMPs have been developed, and demonstrate marked selectivity between different MMPs [132,133,134,135,136]. Studies examining the specificity of a number of MMP inhibitors have demonstrated that the inclusion of chelation moieties did not inherently induce the off-target inhibition of other key metal-containing proteins, such as cyclooxygenase (COX; iron-dependent heme moiety), inducible nitric oxide synthase (iNOS; non-heme iron-dependent enzyme), etc. [137]. In these studies, the dose of the agent administered could be critical in terms of not inducing deleterious side effects. A pertinent example regarding this dose response effect is the use of thiosemicarbazones for cancer treatment [138,139]. In this case, their marked anti-cancer potency necessitates the use of low doses, which do not deplete whole organismal metal ion pools (in particular, iron) to induce overt side effects such as anemia [138,139].

### 8.2. Thiosemicarbazones: Complex Inhibition of MMPs by Multiple Mechanisms

**Figure 4:**
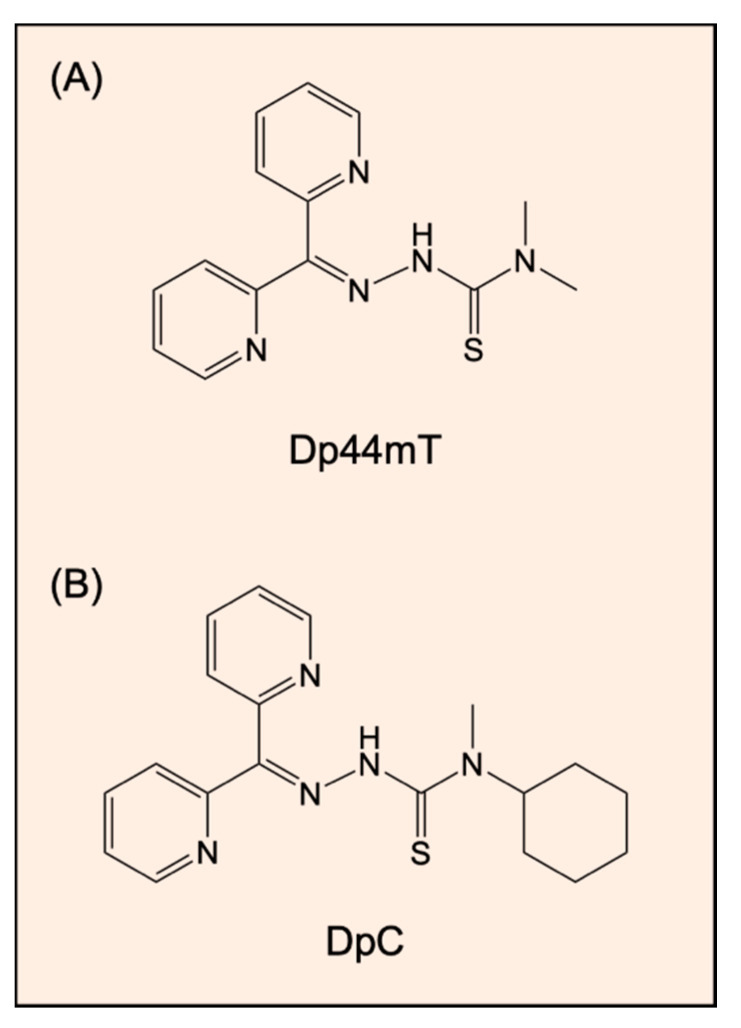
Line drawings of the structures of thiosemicarbazones: (**A**) di-2-pyridylketone 4,4-dimethyl-3-thiosemicarbazone (Dp44mT) and (**B**) di-2-pyridylketone-4-cyclohexyl-4-methyl-3-thiosemicarbazone (DpC). Both agents exert potent and selective anti-cancer activity in a wide variety of cancer models in vitro and in vivo [138,139,140,141,142] via a variety of molecular mechanisms [148,149,150,151,152,153,154,155].

The recently designed thiosemicarbazones di-2-pyridylketone 4,4-dimethyl-3-thiosemicarbazone (Dp44mT; Figure 4A) and di-2-pyridylketone-4-cyclohexyl-4-methyl-3-thiosemicarbazone (DpC; Figure 4B) exert potent and selective anti-cancer activity in a wide variety of cancer models in vitro and in vivo [138,139,140,141,142]. Despite Dp44mT causing the reversible oxidation of heme iron, leading to cardiac met-myoglobin and met-hemoglobin formation in mice, this effect was not observed in mice treated with DpC [143]. In fact, DpC recently entered phase I clinical trials for the treatment of advanced cancer [144]. As metal ion chelators, they induce iron depletion in cells [138,141], but also form a redox active iron or copper complexes within tumor cells that generate reactive oxygen species (ROS; [138,141,145,146,147]). Studies have shown that these agents target and suppress diverse oncogenic signaling pathways, including protein kinase B (AKT), PI3K, RAS, EGFR, HER2, HER3, and c-MET [148,149,150,151,152,153]. This occurs, at least in part, via the ability of Dp44mT or DpC to up-regulate the expression of the metastasis suppressor protein called the N-myc downstream regulated gene 1 (NDRG1) by hypoxia inducible factor 1α-dependent and -independent mechanisms [154,155].

Through the ability of thiosemicarbazones to up-regulate NDRG1 via cellular iron sequestration [154,155], these agents have been demonstrated to inhibit MMP expression. In fact, recent studies by Sharma and associates [146] examining prostate cancer cells demonstrated that NDRG1 regulates the expression and glycosylation of EMMPRIN, which, as discussed above, is a master regulator of MMP activity in tumor cells and tumor-associated fibroblasts [56,156]. Decreased NDRG1 expression resulted in increased EMMPRIN expression, with a concomitant increase in MMPs and invadopodia activity [157]. In these latter studies, a decreased NDRG1 expression resulted in an increase of MMP-2 and MMP-9 activities in HEK293 cells, while for DU145, RWPE, PC3, and LNCaP cells, there was a promotion of MMP-2 activity.

Similar effects of NDRG1 on MMP-2 expression have also been reported whilst examining human gastric adenocarcinoma cells. In these latter studies, the invasive ability of these cells in terms of the matrigel invasion activity and gelatinolytic activity through MMP-2 were increased in *NDRG1* silenced cells, while this activity was reduced upon the re-expression of NDRG1 [158]. Moreover, the induction of MMP-2 by decreasing NDRG1 expression was reported to be mediated through *MT1*-*MMP* that acts selectively on MMP-2 [159]. Of note, MT1-MMP itself is an integral type I transmembrane, multi-domain zinc(II)-dependent endopeptidase involved in extracellular matrix remodeling [89]. Both MMP-2 and MMP-9 play important roles in tumor invasion, degrading the matrix and activating latent TGF-β present in the extracellular space [160]. In summary, as part of the multi-modal anti-metastatic activity of NDRG1 [161,162], this metastasis suppressor decreases MMP expression that is important for invasion.

In addition to NDRG1, Wang and colleagues demonstrated that the NDRG1-inducer Dp44mT also up-regulated NDRG2, with the inhibition of MMP-2 activity being demonstrated in hepatocellular carcinoma cells [163]. Considering that silencing *NDRG2* expression partially abrogated the Dp44mT-induced effect on MMP-2, it was suggested that Dp44mT suppresses MMP-2 activity via NDRG2 up-regulation [163]. Like NDRG1, NDRG2 is known to act as a metastasis suppressor [163,164,165]. Additionally, NDRG2 expression also up-regulates bone morphogenetic protein-4, which inhibits MMP-9 activity in breast tumor cells [166]. In summary, these studies indicate that Dp44mT has impressive properties at the molecular level on at least two members of the NDRG metastasis suppressor family that modulate MMP-2 and -9 expression. This latter effect probably explains, in part, the marked effect of the expression of these metastasis suppressors on inhibiting tumor cell migration, invasion, and metastasis in vivo [154,163,167,168,169].

A recent study by Lim and associates has demonstrated that, in prostate cancer cells, Dp44mT and DpC can induce proteasomal degradation of the androgen receptor (AR) via the up-regulation of c-Jun [153]. This effect leads to the suppression of AR transcription in prostate cancer cells, reducing the expression of PSA, which is an important downstream AR target [153]. Of note, PSA is a member of the KLKs and is also known as KLK-3 [170], and has been demonstrated, in prostate cancer cells, to promote the epithelial mesenchymal transition (EMT) and cell migration by decreasing E-cadherin levels [171]. Therefore, the ability of DpC to inhibit PSA expression could lead to effective anti-metastatic activity against prostate cancer cells [153]. These studies demonstrated that DpC may be more potent against castrate-resistant prostate cancer than the agent Enzalutamide [153], which is widely used in clinics for advanced prostate cancer [172]. This potent activity is due to DpC exerting broad inhibition of both androgen-dependent and -independent AR signaling pathways [153]. In contrast, Enzalutamide only inhibits androgen-dependent AR signaling [172].

Apart from the indirect effect of Dp44mT and/or DpC on PSA and MMP-2, it is well-known that the direct chelation of zinc(II) from the active sites of MMPs may play a critical role in preventing the activity of this enzyme. This is important to consider, as Dp44mT and DpC not only bind iron(II) and copper(II), but also zinc(II) [173,174], and other thiosemicarbazones have been demonstrated to effectively inhibit metalloprotease activity in snake venom [175].

**Figure 5:**
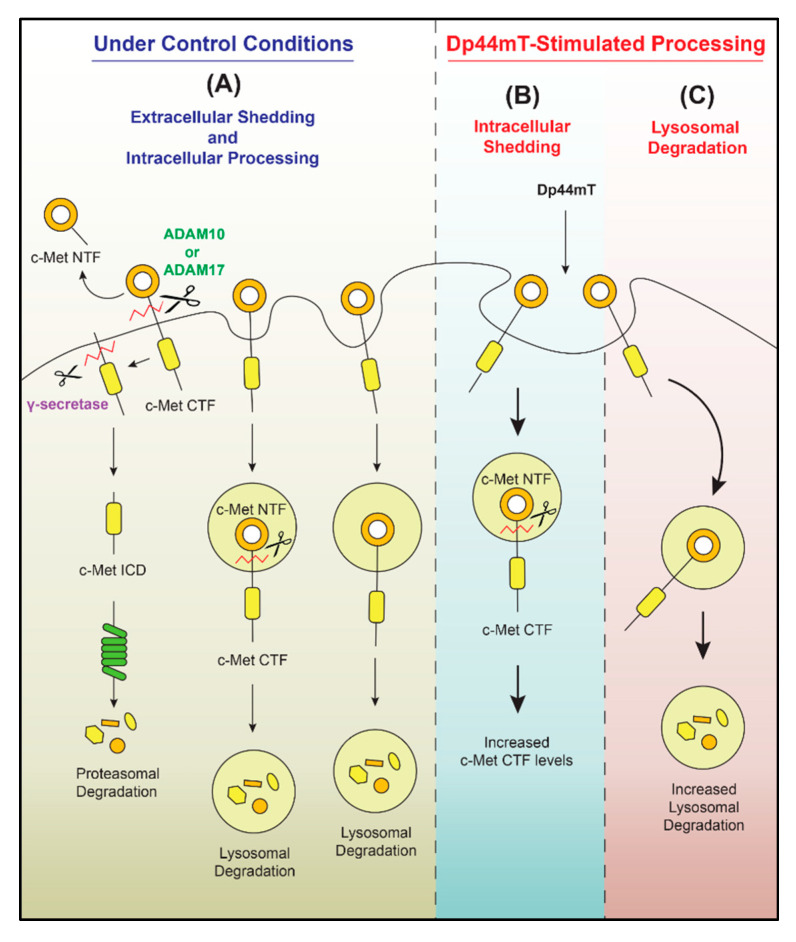
The thiosemicarbazone Dp44mT down-regulates c-MET expression in cancer cells. (**A**) Under control conditions, the c-MET protein undergoes proteolytic cleavage by membrane-bound proteases, such as ADAM-10 and/or -17. This process generates the c-MET *N*-terminal fragment (NTF) that is liberated from the cell and the c-MET *C*-terminal fragment (CTF). The c-MET CTF is then further cleaved by γ-secretase to produce a smaller fragment—the c-MET intracellular domain (ICD)—which is then readily degraded by the proteasome. The cleavage of c-MET could also occur intracellularly, and the c-MET protein is also internalized and degraded by lysosomal activity after the incubation of cells with Dp44mT. Dp44mT enhances (**B**) metalloproteinase-mediated intracellular shedding of the c-MET protein and (**C**) the lysosomal degradation of c-MET. Taken from [152].

As described above, while there is ample evidence for the ability of chelators and thiosemicarbazones to inhibit MMP activity [105], their effects are not simple and can lead to the enhancement of MMP activity. In fact, a recent study from our laboratory demonstrated that the expression of the oncoprotein c-MET could be down-regulated upon the incubation of multiple tumor cell types in vitro with Dp44mT or DpC [152]. This decrease in c-MET occurred by multiple mechanisms, including lysosomal degradation, but also increased metalloprotease-mediated cleavage, resulting in increased generation of the c-MET *C*-terminal fragment (Figure 5). The broad metalloprotease inhibitors EDTA and batimastat partially prevented the Dp44mT-mediated down-regulation of c-MET. In contrast, the ADAM inhibitor TIMP metallopeptidase inhibitor 3 (TIMP-3) had no such effect, suggesting c-MET cleavage by another metalloprotease [152]. The down-regulation of c-MET by thiosemicarbazones led to a decrease in the phosphorylation of Gab1, which is a major downstream effector of the c-MET pathway, indicating the suppression of c-MET oncogenic activity [152]. Therefore, the effects of metal ion chelation upon cancer cells can be complex, with these effects probably being due to their multi-targeted activity, as metal ions are critical for a variety of key metabolic processes [176]. Nonetheless, thiosemicarbazones and other chelators result in marked anti-oncogenic activity against a broad variety of tumor cells in vitro [177,178,179] and in vivo [151,180,181].

Collectively, the studies described above indicate that the critical catalytic zinc(II) ion in the active site of MMPs is an important molecular target for the design of further chelating agents. Moreover, it has been demonstrated that the thiosemicarbazone group of ligands have complex and potent inhibitory effects on the extracellular activity of a number of key proteases that play critical roles in cancer progression.

## 9. Conclusions

Extracellular proteases play critical roles in a variety of physiological and pathological processes, including the metastasis of tumor cells. Therefore, they have become prime molecular targets for newly developed therapeutics, and this underscores the importance of understanding the molecular mechanisms that control their expression and activity. Considering this, it is not surprising that key metastasis suppressor proteins, such as NDRG1 and NDRG2, have been demonstrated to down-regulate MMPs. Agents which up-regulate NDRG1 expression, including chelators of the thiosemicarbazone class and others, can down-regulate MMP expression via indirect mechanisms. However, these compounds can also directly decrease MMP activity by binding zinc(II) that is required in the active site and is essential for enzymatic activity. Considering the recent discoveries of the indirect effects of thiosemicarbazones on the expression and activity of extracellular proteases, further investigation in this area could lead to a novel therapeutic strategy in terms of the tailored clinical application of these agents.

## Figures and Tables

**Table 1:** Diverse matrix metalloproteases (MMPs) with either tumor-promoting or -suppressing functions in different types of cancer.

MMPs	Function	Cancer Type
MMP-1	1. Promotes cancer cell proliferation, migration and invasion via cleavage of protease-activated receptor1 2. Promotes multidrug resistance	1. Breast cancer 2. Breast cancer
MMP-2	1. Promote cancer cell migration by interaction with collagen 2. Promotes tumor invasiveness via ECM degradation 3. Promotes tumor growth by inducing vessel maturation and function	1. Fibrosarcoma 2. Breast cancer 3. Murine brain tumor
MMP-3	1. Promotes metastasis 2. Promotes tumor formation 3. Inhibits angiogenesis by producing angiostatin and endostatin through cleavage of plasminogen and type VIII collagen	1. Mouse mammary tumor 2. Mouse mammary tumor 3. Breast cancer
MMP-7	1. Promotes cancer cell migration and invasion 2. Promotes tumor formation 3. Promotes EMT	1. Colorectal carcinoma 2. Colorectal carcinoma 3. Prostate adenocarcinoma
MMP-8	1. Protective role against carcinogens by potentially cleaving inflammatory mediators 2. Inhibits cancer cell invasion and migration by modulating gene, protein expression, including decrease in VEGF-C; decreases ligand binding of β1 integrin 3. Promotes cancer cell invasion and migration by up-regulating TGF-β1 expression through activation of PI3K/AKT/RAC1 pathway 4. Inhibits cancer cell proliferation 5. Promotes cancer cell proliferation 6. Anti-metastatic role 7. Worsen the prognosis	1. Papilloma 2. Oral tongue squamous cell carcinoma, prostate cancer 3. Hepatocellular carcinoma (HCC) 4. Lung cancer, breast cancer 5. Osteosarcoma 6. Melanoma, breast cancer 7. HCC, ovarian cancer, colorectal cancer
MMP-9	1. Promote metastasis 2. Inhibits angiogenesis by cleaving IGFBP-2	1. Triple negative breast cancer, gastric adenocarcinoma 2. Astrocytoma
MMP-10	1. Promotes angiogenesis 2. Inhibits apoptosis 3. Promotes cancer cell invasion 4. Promotes stemness	1. Cervical tumor 2. Cervical tumor 3. Head and neck cancer 4. Ovarian cancer
MMP-11	1. Inhibits apoptosis via activation of p42/p44 MAPK 2. Promotes cancer cell migration and invasion 3. Suppresses metastasis	1. Breast cancer 2. Erα- breast cancer, colon carcinoma 3. Mouse mammary tumor virus-ras tumor
MMP-12	Promotes tumor formation	Bronchioalveolar adenocarcinoma
MMP-13	Promotes angiogenesis via stimulation of ERK-FAK signaling pathway and VEGF-A secretion	Head and neck squamous cell carcinoma
MMP-14	Induces chromatin instability by cleaving pericentrin	Glioma, breast cancer and colon adenocarcinoma
MMP-17	1. nvolved in tumor cell intravasation by enlarging intratumoral blood vessels and augmenting vessel leakage 2. Modulates 65 miRNAs involved in tumor formation and progression 3. Drives cancer cell proliferation through CDK4 and EGFR activation, retinoblastoma protein inactivation	1. Breast cancer 2. Breast cancer 3. Breast cancer
MMP-19	1. Promotes EMT, migration and invasion 2. Tumor suppressive and anti-angiogenic functions	1. Non-small cell lung cancer, melanoma, glioblastoma 2. Nasopharyngeal carcinoma
MMP-26	1. Suppresses tumor progression by cleaving ERβ 2. Proapoptotic role	1. Breast cancer 2. Prostate cancer
MMP-28	1. Induces EMT and cell migration via proteolytic activation of TGF-β, elevating ZEB levels 2. Promotes cell migration, invasion and metastasis via Notch3 signaling	1. Lung carcinoma, HCC 2. HCC
